# Current Understanding of Circulating Tumor Cells – Potential Value in Malignancies of the Central Nervous System

**DOI:** 10.3389/fneur.2015.00174

**Published:** 2015-08-10

**Authors:** Lukasz A. Adamczyk, Hannah Williams, Aleksandra Frankow, Hayley Patricia Ellis, Harry R. Haynes, Claire Perks, Jeff M. P. Holly, Kathreena M. Kurian

**Affiliations:** ^1^Department of Cellular Pathology, North Bristol NHS Trust, Bristol, UK; ^2^Brain Tumor Research Group, Institute of Clinical Neuroscience, North Bristol NHS Trust, Bristol, UK; ^3^IGF and Metabolic Endocrinology Group, School of Clinical Sciences, North Bristol NHS Trust, University of Bristol, Bristol, UK

**Keywords:** circulating tumor cells, glioblastoma multiforme, glioma, liquid biopsy, epithelial–mesenchymal transition

## Abstract

Detection of circulating tumor cells (CTCs) in the blood via so-called “liquid biopsies” carries enormous clinical potential in malignancies of the central nervous system (CNS) because of the potential to follow disease evolution with a blood test, without the need for repeat neurosurgical procedures with their inherent risk of patient morbidity. To date, studies in non-CNS malignancies, particularly in breast cancer, show increasing reproducibility of detection methods for these rare tumor cells in the circulation. However, no method has yet received full recommendation to use in clinical practice, in part because of lack of a sufficient evidence base regarding clinical utility. In CNS malignancies, one of the main challenges is finding a suitable biomarker for identification of these cells, because automated systems, such as the widely used Cell Search system, are reliant on markers, such as the epithelial cell adhesion molecule, which are not present in CNS tumors. This review examines methods for CTC enrichment and detection, and reviews the progress in non-CNS tumors and the potential for using this technique in human brain tumors.

## Introduction

Detection of circulating tumor cells (CTCs) is of current great interest in central nervous system (CNS) malignancies because of recent intriguing reports, suggesting that cells from a proportion of patients with glioblastoma multiforme (GBM) may be detectable in the bloodstream (1–3). Outwit the CNS field, detection of CTCs represents a promising non-invasive technique to facilitate early diagnosis and monitoring tumor biology evolution, which is underlined by over 500 studies, registered internationally involving CTCs (4–6). The potential of the CTC approach was highlighted in an early study by Ross et al. who described peripheral blood contamination by free floating cells of metastatic breast cancer in patients receiving autologous stem cell transplants (7). Due to the scarcity of CTCs in the blood the interest in this field initially shifted to enhancement of cell detection and identification using techniques, such as immunomagnetic labeling followed by polymerase chain reaction (PCR) (8, 9). RT-PCR and qPCR are widely used today as standards of CTC identification (10) although both methods are highly sensitive and suffer from a false positive rate due to the presence of contaminating cells (9).

Circulating tumor cells have now been described in most common carcinomas including breast, prostate, and colorectal carcinoma (11, 12), and most recently in CNS malignancies (13–17). There is evidence that CTC count has prognostic validity in breast cancer (18) and in particular has been related to progression-free survival (PFS) and overall survival (OS) in patients with metastatic disease (19).

At present, the only CTC detection platform to receive validation by the Food and Drug Administration of the United States of America is the CellSearch^®^ system (Veridex, Raritan, NJ, USA) (20), which is a robust platform but not without its limitations, discussed further in this review. Currently, more advanced methods, e.g., CTC-chip and the EPISPOT allow isolation of still viable tumor cells enabling more detailed analysis (21, 22).

Expanding research in this field has uncovered biological dynamics of CTCs along the metastatic pathway (23) and uncovered tumor subtypes namely stem cells and disseminated tumor cells (DTCs) (24). DTCs are a CTC subpopulation found in bone marrow which may act as a dormant reservoir of malignant disease (4, 25, 26) and it is suggested that their prognostic value might be equal to CTCs (4, 24). A study by Baccelli et al. suggested a subpopulation of CTCs displaying CD44 cancer stem cell and bone homing marker, CD47, which inhibits phagocytosis and MET (a hepatocyte growth factor receptor); these have been postulated to reflect the promotion of metastatic and invasive activity (27).

## Dissemination of Malignancy

Initial stages of potential metastatic tumor spread begin with a heterogenous population of malignant cells where the dynamic changes in the tumor cell genome may give rise to metastasis induction, followed by progression and virulence (28). The development of metastatic disease has been divided into several stages, each characterized by specific genomic, epigenomic, and phenotypic alterations: persistence of proliferation-promoting signals, evasion of growth suppressors, resistance to cell death enabling replicative immortality, promotion of angiogenesis, and initiation of the invasive and metastatic process (29). Studies have demonstrated that as few as 0.01% of circulating cancer cells develop into secondary tumors with oxygenation, pH, nutrient supply, and inflammatory response constantly influencing this process (28). It is now thought that in order to enter the circulation, epithelial tumor cells may undergo epithelial–mesenchymal transition (EMT) (30). Primary epithelial malignant cells may putatively undergo transdifferentiation to a mesenchymal genotype with intermediate epithelial–mesenchymal forms present (4, 23, 25, 26, 30, 31). Furthermore, it has been postulated that to exit the circulatory system, CTCs may in fact undergo a “reverse” mesenchymal–epithelial transition (4), suggesting that the most effective CTCs are probably of an intermediate phenotype. EMT is thought to have an origin in embryogenesis when bulk migration of developing cells occurs through compact tissue stroma and there may be upregulation of many factors including (TGFβ), WNT, platelet-derived growth factor (PDGF), and interleukin-6 (IL-6) (30). While cells undergo EMT they gradually lose their epithelial markers, i.e., E-cadherin, claudin, and plakoglobin (23, 30) and acquire mesenchymal markers, such as fibronectin, cadherin 2, and serine proteinase inhibitor-1 (SERPIN 1) (32).

More recent evidence suggests that in addition to single CTCs, tumor fragments are also represented in the blood as microemboli containing stromal fibroblasts, leukocytes, and platelets (33) creating a “floating” microenvironment. These micro-fragments have been shown to evade anoikis and elimination by the immune system in the bloodstream (30, 33, 34) and promote adhesion and tissue invasion at secondary sites (30, 33, 35). Uppal et al. explored this mechanism by showing that aspirin may disrupt adherence of tumor microemboli at distant tissues (36). CTCs are relatively rare with approximately 1 CTC per 10^5^–10^8^ white blood cells (26). CTCs are phenotypically thought to be a heterogeneous population, each cell showing variable expression of biomarkers (37). However, the epithelial cell adhesion molecule (EpCAM) is a 30–40 kDa transmembrane glycoprotein commonly expressed not only on epithelioid CTCs but also on a proportion of white cells (38). It has become the target molecule for cell selection and enumeration of various detection systems primarily focused on epithelial malignancies (20, 21, 39–45).

In an optimal theoretical model, CTCs should express biomarkers not detected on other intrinsic cells in the bloodstream and not lost in the mesenchymal and circulating cell transition (46). They can be divided according to their function into prognostic, pharmacodynamic, predictive, surrogate, and monitoring biomarkers (5). The extracted cell should remain viable to allow *post hoc* molecular analysis (4, 46) and acquisition of good quality DNA rich material assures more efficient molecular identification of cells (47).

## CTC Enrichment and Detection

Numerous techniques of CTC identification can be divided into broad groups according to methods of cell enrichment and cell detection, which can be used in various combinations (26, 33, 48). The most commonly shared principles of enrichment are antibody mediated or physical methods followed by secondary immunohistochemical enumeration and/or subsequent genetic analysis (33).

The CellSearch^®^ platform utilizes EpCAM labeled CTC enrichment using antibody-coated magnetic beads and labeling with fluorescent-coated antibodies against cytokeratin together with 4′,6-diamidino-2-phenylindole (DAPI) nuclear coating (19, 20). Although widely used, it is recognized that EpCAM-based enrichment suffers from limitations, such as relatively low sensitivity and purity, partly due to the presence of EpCAM negative tumor cells (38, 49). For example, cells expressing CD45+ EpCAM+ were demonstrated to represent a macrophage population that may be a source of false positivity (50).

Enrichment selection methods using the anti-EpCAM antibody have been evolving through the introduction of microfluidic chips (CTC-chip, CTC-iChip^®^, Herringbone Chip, etc.) and of variations to known immunomagnetic and flow cytometry techniques (39, 45). Novel models have described the use of an antibody-coated intravenous wire, which is inserted directly into a vein (40). By directly exposing the probe to a constant large-volume flow of blood this method increases the probability of capturing CTC thereby addressing the issue of their very low concentration. Fisher et al. incorporated leukapheresis together with CellSearch^®^ to address this issue (51). A variety of physical property-based enrichment methods have also been introduced, such as dielectrophoretic field flow fractionation (DEP-FFF) (52), ISET^®^ (53), or Dean flow fractionation (54), some of which may allow cell culture of retrieved CTCs.

**Figure 1:**
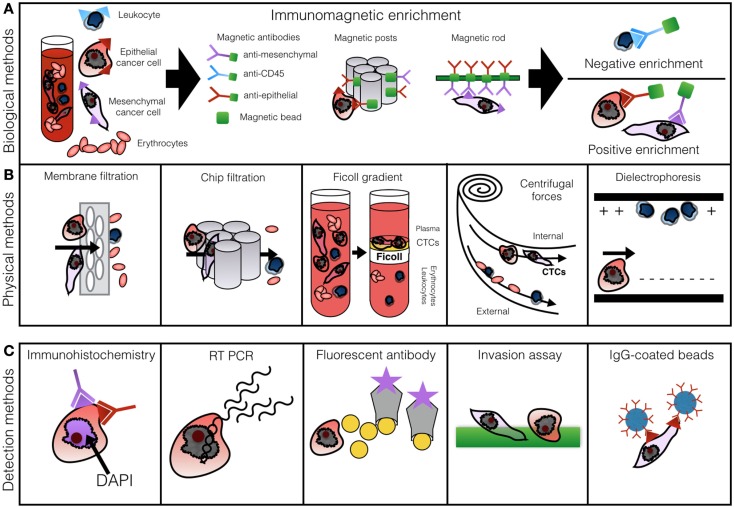
**Row (A) demonstrates methods of biological CTC enrichment using magnetically labeled antibodies captured in a magnetic chamber or by posts or rods**. Row **(B)** illustrates physical enrichment methods: membrane filtration, microfluidics, Ficoll gradient centrifugation, Dean drag forces separation, and dielectrophoresis. Row **(C)** outlines the most common principles of cell enumeration: IHC, RT-PCR, fluorescent antibody labeling, invasion assay, or antibody-coated beads. Based on Alix-Panabieres and Pantel (46) with permission.

Figure 1 presents the most commonly described methods of CTC enrichment and detection. A standard 7.5–10 ml blood sample is processed within 2 h of withdrawal. Biological enrichment incorporates anti-epithelial, leukocytic, or mesenchymal antibodies labeled by a magnetic particle or affixed to a post or a rod (20, 39, 55). Positive enrichment relies on selective capture of CTCs, while negative enrichment through labeling of CD45 filters out cells which express leukocytic markers (22).

Modified immunomagnetic methods can achieve high degree of CTC purification and to allow downstream analysis, while some techniques preclude cell culture of retrieved CTCs. Combination of techniques, such as MoFloXDP cell sorting, with qPCR allows high-throughput analysis through single cell-array based comparative genomic hybridization (56, 57). In a related method, the AdnaTest utilizes double EMA and EpCAM magnetic bead enrichment followed by RT-PCR multigene panel (56). Microfluidic on-chip methods have been developed offering a single device solution and efficient analysis pairing immunomagnetic enrichment with IHC or PCR (21, 41–43, 58). Certain chips offer single cell high-throughput analysis through physical enrichment taking advantage of cell size and deformity, again with biological properties preserved (59). Others combine filter-based methods with on-filter immunofluorescence (60). The micro-Hall detector (μHD) chip enriches CTCs with immunomagnetic nanoparticles allowing up to 10^7^ cell/min analysis preserving antigeneity permitting the use of bespoke imunoprofiles (61). An additional advantage of this approach is the ability to analyze unpurified samples which reduces processing time (61). A different nanoparticle method uses gold particles with single strand DNA, which bind to intracellular mRNA in live cells. The entry of nanoparticles into cells does not induce cell death preserving the isolated CTCs for downstream phenotyping (62).

Apart from CTCs, circulating tumor DNA (ctDNA) fragments offer an attractive quantification tool through digital PCR assay and targeted deep sequencing (63), which is based on the samples obtained from 30 patients with metastatic breast cancer. Dawson et al. found ctDNA to be of superior prognostic value to Ca15.3 and CTCs (63). However, the biological properties of CTCs have the potential of specific tumor phenotyping (63). Investigation into other uses, such as diagnosis of malignancies of unknown primaries, offers another interesting potential use of this methodology (64).

## CTCs as Predictors of Survival in Non-CNS Malignancies

In 2007, the American Society of Clinical Oncology issued treatment guidelines, which did not recommend routine detection of CTCs in breast cancer patients in part due to a lack of evidence base as to their prognostic utility (65). However, numerous publications including meta-analyses have emerged since which may be addressed in updated guidelines. Following a recent international multi center study, there is now substantial evidence that detection of five or more CTCs in the blood of breast cancer patients leads to decreased OS and PFS at different stages of follow-up (66). A recent meta-analysis by Zhang et al. found that CellSearch^®^ enrichment combined with RT-PCR was superior in predicting PFS, while prediction of OS was similar regardless of the methods used (67). Prediction of OS was most significant when CTCs were measured at cancer baseline compared with other stages of disease (67). CTCs have been found in patients in all clinical stages including patients with T1 and T2 operable disease regardless of tumor stage, grade, lymph node, or receptor status (68). Some authors suggested a single detected CTC carries higher disease progression risk in non-metastatic chemosensitive (69) and locally advanced (70) breast cancer. Furthermore, in a related study by Pierga et al. showed predictive significance even if detected at a rate of ≥1 CTCs (71).

Presently, ongoing trials are focusing on particular stages of treatment and analyze methods of CTC detection in metastatic breast cancer (CTC-EMT, CTC-CEC-AND), provide input on clinical aspect relating to cost-effectiveness (STIC CTC), and evaluate patients at specific clinical points providing information on prognostication and treatment guidance (Detect III, CirCe01, Treat CTC, COMETI P2) (25). In aggressive triple negative cancers, baseline and early follow-up measurement of CTCs identifies groups of patients with higher tumor chemoresistance (TBCRC 019) (72).

The LANDSCAPE trial investigated patients with metastatic disease to the brain comparing CTC levels before treatment and after lapatinib and capecitabine at 3 weeks in Her2 positive tumors (71). The results were compared with levels of soluble serum biomarkers. The authors found CTCs predict treatment response more accurately avoiding post treatment biomarker spike (71). Interestingly, CTCs occurred less frequently in patients with isolated brain metastases presumably due to properties of the blood–brain barrier (71, 73). Metastatic breast cancer cells variably express EpCAM but may have a more distinct phenotype HER2+/EGFR+/HPSE+/Notch1+ (74). An additional, potentially useful marker, aldehyde dehydrogenase 1 (ALDH1), is of interest as cells lacking this enzyme are unable to form tumors (75).

Second to breast, CTCs have been commonly utilized in prostate carcinoma with the same CTC cut-off of five or more CTCs found to be correlating significantly with prognosis (76). The predictive value of CTCs was described in castration resistant prostate cancer following prostate-specific antigen (PSA) and lactate dehydrogenase (LDH) serum levels (77). Although the CellSearch^®^ system appears to be the method of choice in prostate cancer, more specific markers may be necessary, such as cadherin-11, which is expressed not only on prostate cells and osteoblasts but also on prostate cancer cells exhibiting EMT (78).

Colorectal cancer CTCs traverse the portal circulation with a proportion of the cells being filtered by the liver (79). In colorectal cancer, a value of 3 CTCs or above has commonly been used (80). A large study from the USA showed an independent prognostic effect of CTC counts regardless of serum carcinoembryonic antigen (CEA) levels (81). However, measurement of CTCs prior to resection of liver metastases does not appear to show prognostic effect (82). Colorectal CTCs have been demonstrated to show similar KRAS and BRAF gene status to the primary tumor with 68–100% concordance (5, 83). This may allow identification of patients more likely to be resistant to EGFR inhibitors, but methods of more effective enrichment are required to prevent false negative mutation results (83).

Several studies emerged which confirmed the presence of CTCs in both small cell and non-small cell lung carcinomas. Hou et al. found 85% of patients with confirmed cancer had detectable circulating cells at baseline (84). These authors also devised a bespoke method of establishing the best predictive CTC cut-off, arguing that the values should vary according to individual biological properties of cancers (84). Lung cancer CTCs were also shown to be suitable for EGFR receptor status analysis (85).

To date, CTCs have also been confirmed in ovarian, esophageal, urothelial, pancreatic, head and neck (13–17) carcinomas using a mixture of CellSearch^®^ platform paired with PCR or microfluidic technologies. CTC thresholds range from 1 to 5 CTCs as cut-off but studies are conducted on small groups and require validation.

## Potential Value of CTCs in CNS

Despite the fact that systemic metastases are rare in GBM, a few recent studies have successfully isolated CTCs from peripheral blood of both primary and recurrent adult GBM and diffuse glioma, which could yield great potential for disease monitoring to guide treatment (see Table 1). A key issue is finding an appropriate CNS biomarker to identify the CTCs, because CNS malignancies do not express EpCAM, unlike many epithelial malignancies, which commonly metastasize (3, 86). In a large study by Müller et al., CTCs were identified in 29/141 (20.6%) of GBM patients using physical separation methods (Ficoll gradient) followed by immunostaining for glial fibrillary acidic protein (GFAP) (3). In this case, the use of GFAP for CTC identification was supported by its absence in control participants, and the presence of EGFR amplifications in the tumor cells isolated using GFAP (3). The mobilization of CTCs into the peripheral blood, which still maintains EGFR amplifications supports the hypothesis that they do maintain growth potential (3).

**Table 1:** **Summary of studies attempting to isolate CTCs from patients with high-grade gliomas**.

Author	Cell enrichment	Cell characterization	Results	Limitations
**PUBLICATIONS POSITIVELY IDENTIFYING CTCs IN BRAIN TUMOR PATIENTS**				
Müller et al. (3)	MNCs isolated by Ficoll density gradient centrifugation	GBM patients	*n* = 29/141	Low detection rate
	Cytospins prepared from MNCs GFAP positive single cells isolated by micromanipulation	Chromogenic and fluorescent IHC (GFAP, CD45, EGFR)	Observed association between EGFR amplification and release of CTCs Common genomic aberrations in CTCs and GBM tumors
		Further characterization of CTCs and associated tumor		
		Comparative genomic hybridization	
		Sequence analysis		
		FISH	
Sullivan et al. (87)	Blood processed through a CTC-iChip^®^ (magnetically tagged CD45 and CD16)	GBM patients	*n* = 26/87	Limited dataset
	IF-guided single-cell micromanipulation used to isolate single CTCs (EGFR, MET and CDH11)	IHC glioma marker panel [SOX2, Tubulin, beta-3, EGFR, A2B5 and c-MET (STEAM)]	RNA-ISH demonstrated an enrichment for mesenchymal transcripts and a reduction of neural differentiation markers	Could not determine whether surgical or radiation induced disruption of the blood–brain barrier enhances CTC dissemination
		FISH used to determine EGFR gene amplification in CTCs from known amplified cases		
		Further CTC characterization by qRT-PCR and dual color RNA-ISH assay	
MacArthur et al. (88)	Blood samples centrifuged in OncoQuick tubes	High-grade glioma patients	*n* = 8/11 pre-radiotherapy	Limited pilot data
		Incubated with a telomerase-responsive adenoviral probe (via GFP expression) Secondary Immunofluorescence (Nestin and EGFR)	*n* = 1/8 post-radiotherapy	Need more serial measurements throughout the treatment and disease course for each patient
			EGFR amplification in CTCs correlates with solid tumors	Telomerase is elevated in other tumor histologies
**PUBLICATIONS NEGATIVELY IDENTIFYING CTCs IN BRAIN TUMOR PATIENTS**				
Böhm et al. (89)	Total cellular RNA extracted from whole blood using the QIAmp RNA blood mini kit (Qiagen)	High-grade astrocytoma and GBM patients	*n* = 0/20	Sample size
		RT-qPCR assay for the detection of mRNA encoding GFAP and B2M (positive control)		Insufficient technology
Martens et al. (90)	Cytocentrifugation	Astrocytoma patient	*n* = 0/1	Only one patient and sample tested
		Chromogenic and fluorescent IHC (GFAP)		Insufficient technology

Moreover, authors from Massachusetts Institute of Technology recently published a set of biomarkers found on CTCs with the use of a CTC-iChip^®^ (87). The STEAM panel consisted of sex determining region Y-box 2 (SOX2), tubulin beta-3, EGFR, A2B5, and c-Met and found specifically on high-grade glioma cells (87). Circulating glioma tumor cells were found to harbor elevated SERPINE1, TGFB1, TGFBR2, and vimentin, which are associated with an aggressive mesenchymal phenotype (87). The authors suggest that there may be a subset of mesenchymal cells present in disseminated GBM that have the ability to invade the vascular system and proliferate outside the brain as systemic lesions (87).

An interesting approach used in the pilot study by MacArthur et al. identified CTCs with an adenoviral telomerase-responsive probe that consisted of the expression cassette for green fluorescent protein (GFP) as well as the hTERT promoter driving expression of E1A and E1B for viral replication and amplification of the GFP signal that can detect the increased telomerase activity in the CTCs following physical separation with OncoQuick tubes (88). This was combined with immunofluorescence for GFAP and nestin which helped to delineate the glial origin of the CTCs (88). The MacArthur study identified circulating glioma cells in 8 of 11 (72%) pre-radiotherapy high-grade glioma patients, compared with 1 of 8 (12%) in the post-radiotherapy cohort, demonstrating the ability of the liquid biopsy to identify patients at risk of recurrence/with high tumor burdens (88).

There is in addition a potential of the use of CTCs in the identification of patients with a phenomenon known as pseudoprogression – when the radiological features mimic tumor recurrence – but, in fact, the tumor may be undergoing a non-malignant inflammatory change (2).

Cerebrospinal fluid (CSF) is also a potential source for glioma CTCs biomarkers; however, this has not yet been evaluated in the literature to date (2, 91, 92).

## Conclusion

Detection of CTCs via so-called “liquid biopsies” carries enormous clinical potential in CNS malignancies and requires urgent further research. To date, studies in non-CNS malignancies, particularly in breast cancer, show increasing reproducibility of detection methods for these rare tumor cells in the circulation. However, no method has yet received full recommendation to use in clinical practice, in part because of lack of a sufficient evidence base regarding clinical utility.

In CNS malignancies, one of the main challenges is finding a suitable biomarker for identification of these cells, because automated systems, such as the widely used Cell Search system, are reliant on markers, such as EpCAM, which are not present in CNS tumors. There are ongoing promising initial studies which have identified CTCs in the peripheral blood of glioma patients using physical separation techniques followed by IF for markers, such as GFAP, nestin, and a telomerase promoter-based assay, or iCHIP using the STEAM panel that consisted of SOX2, tubulin beta-3, EGFR, A2B5, and c-Met.

## Author Contributions

LA: manuscript research, writing, revision, and figure design; HW: table, manuscript review, and revision; AF: manuscript review and revision; HE: manuscript review and revision; HH: manuscript review and revision; CP: manuscript review and revision; JH: manuscript review and revision; KK: manuscript writing, review, and revision.

## Conflict of Interest Statement

The authors declare that the research was conducted in the absence of any commercial or financial relationships that could be construed as a potential conflict of interest.

## Abbreviations

BBB, blood–brain barrier; CNS, central nervous system; CTC, circulating tumor cells; ctDNA, circulating tumor DNA; DAPI, 4′,6-diamidino-2-phenylindole; DTCs, disseminated tumor cells; EMT, epithelial–mesenchymal transition; EpCAM, epithelial cell adhesion molecule; FISH, fluorescent *in situ* hybridization; GBM, glioblastoma multiforme; GFAP, glial fibrillary acidic protein; hGBM, primary glioblastoma multiforme cells; IF, immunofluorescence; IHC, immunohistochemistry; ISH, *in situ* hybridization; MNC, mononuclear cells; OS, overall survival; PCR, polymerase chain reaction; PFS, progression-free survival; QPCR, quantitative PCR; RT-PCR, real-time PCR.

## References

[1] Best MG Sol N Zijl S Reijneveld JC Wesseling P Wurdinger T. Liquid biopsies in patients with diffuse glioma. Acta Neuropathol (2015) 129(6):849–65. 10.1007/s00401-015-1399-y 25720744 PMC4436687

[2] Kros JM Mustafa DM Dekker LJ Sillevis Smitt PA Luider TM Zheng P-P. Circulating glioma biomarkers. Neuro Oncol (2014) 17(3):343–60. 10.1093/neuonc/nou207 25253418 PMC4483097

[3] Müller C Holtschmidt J Auer M Heitzer E Lamszus K Schulte A Hematogenous dissemination of glioblastoma multiforme. Sci Transl Med (2014) 6:247ra101. 10.1126/scitranslmed.3009095 25080476

[4] Bednarz-Knoll N Alix-Panabières C Pantel K. Clinical relevance and biology of circulating tumor cells. Breast Cancer Res (2011) 13:228. 10.1186/bcr2940 22114869 PMC3326546

[5] De Gramont A Watson S Ellis LM Rodón J Tabernero J de Gramont A Pragmatic issues in biomarker evaluation for targeted therapies in cancer. Nat Rev Clin Oncol (2014) 12(4):197–212. 10.1038/nrclinonc.2014.202 25421275

[6] ClinicalTrials.gov [Internet] (2015) [cited 2015 May 3]. Available from: https://clinicaltrials.gov/ct2/results?term=circulating+tumour+cells&Search=Search

[7] Ross AA Cooper BW Lazarus HM Mackay W Moss TJ Ciobanu N Detection and viability of tumor cells in peripheral blood stem cell collections from breast cancer patients using immunocytochemical and clonogenic assay techniques. Blood (1993) 82:2605–10. 8219214

[8] Racila E Euhus D Weiss AJ Rao C McConnell J Terstappen LW Detection and characterization of carcinoma cells in the blood. Proc Natl Acad Sci U S A (1998) 95:4589–94. 10.1073/pnas.95.8.4589 9539782 PMC22534

[9] Ghossein RA Bhattacharya S Rosai J. Molecular detection of micrometastases and circulating tumor cells in solid tumors. Clin Cancer Res (1999) 5:1950–60. 10473071

[10] Jiang WG Martin TA Mansel RE. Molecular detection of micro-metastasis in breast cancer. Crit Rev Oncol Hematol (2002) 43:13–31. 10.1016/S1040-8428(01)00181-0 12098605

[11] Wang ZP Eisenberger MA Carducci MA Partin AW Scher HI Ts’o PO. Identification and characterization of circulating prostate carcinoma cells. Cancer (2000) 88:2787–95. 10.1002/1097-0142(20000615)88:12<2787::AID-CNCR18>3.0.CO;2-2 10870062

[12] Molnar B Ladanyi A Tanko L Sréter L Tulassay Z. Circulating tumor cell clusters in the peripheral blood of colorectal cancer patients. Clin Cancer Res (2001) 7:4080–5. 11751505

[13] Aktas B Kasimir-Bauer S Heubner M Kimmig R Wimberger P. Molecular profiling and prognostic relevance of circulating tumor cells in the blood of ovarian cancer patients at primary diagnosis and after platinum-based chemotherapy. Int J Gynecol Cancer (2011) 21:822–30. 10.1097/IGC.0b013e318216cb91 21613958

[14] Reeh M Effenberger KE Koenig AM Riethdorf S Eichstädt D Vettorazzi E Circulating tumor cells as a biomarker for preoperative prognostic staging in patients with esophageal cancer. Ann Surg (2015) 261(6):1124–30. 10.1097/SLA.0000000000001130 25607767

[15] Gallagher DJ Milowsky MI Ishill N Trout A Boyle MG Riches J Detection of circulating tumor cells in patients with urothelial cancer. Ann Oncol (2008) 20:305–8. 10.1093/annonc/mdn627 18836088

[16] Görner K Bachmann J Holzhauer C Kirchner R Raba K Fischer JC Genetic analysis of circulating tumor cells in pancreatic cancer patients: a pilot study. Genomics (2015) 106(1):7–14. 10.1016/j.ygeno.2015.02.003 25812950

[17] Grisanti S Almici C Consoli F Buglione M Verardi R Bolzoni-Villaret A Circulating tumor cells in patients with recurrent or metastatic head and neck carcinoma: prognostic and predictive significance. PLoS One (2014) 9:e103918. 10.1371/journal.pone.0103918 25105871 PMC4126745

[18] Gaforio J-J Serrano M-J Sanchez-Rovira P Sirvent A Delgado-Rodriguez M Campos M Detection of breast cancer cells in the peripheral blood is positively correlated with estrogen-receptor status and predicts for poor prognosis. Int J Cancer (2003) 107:984–90. 10.1002/ijc.11479 14601059

[19] Cristofanilli M Hayes DF Budd GT Ellis MJ Stopeck A Reuben JM Circulating tumor cells: a novel prognostic factor for newly diagnosed metastatic breast cancer. J Clin Oncol (2005) 23:1420–30. 10.1200/JCO.2005.08.140 15735118

[20] Riethdorf S Fritsche H Müller V Rau T Schindlbeck C Rack B Detection of circulating tumor cells in peripheral blood of patients with metastatic breast cancer: a validation study of the CellSearch system. Clin Cancer Res (2007) 13:920–8. 10.1158/1078-0432.CCR-06-1695 17289886

[21] Nagrath S Sequist LV Maheswaran S Bell DW Irimia D Ulkus L Isolation of rare circulating tumour cells in cancer patients by microchip technology. Nature (2007) 450:1235–9. 10.1038/nature06385 18097410 PMC3090667

[22] Alix-Panabières C. EPISPOT assay: detection of viable DTCs/CTCs in solid tumor patients. Recent Results Cancer Res (2012) 195:69–76. 10.1007/978-3-642-28160-0_6 22527495

[23] Thiery JP Acloque H Huang RY Nieto MA Epithelial-mesenchymal transitions in development and disease. Cell (2009) 139:871–90. 10.1016/j.cell.2009.11.007 19945376

[24] Balic M Lin H Young L Hawes D Giuliano A McNamara G Most early disseminated cancer cells detected in bone marrow of breast cancer patients have a putative breast cancer stem cell phenotype. Clin Cancer Res (2006) 12:5615–21. 10.1158/1078-0432.CCR-06-0169 17020963

[25] McInnes LM Jacobson N Redfern A Dowling A Thompson EW Saunders CM. Clinical implications of circulating tumor cells of breast cancer patients: role of epithelial-mesenchymal plasticity. Front Oncol (2015) 5:42. 10.3389/fonc.2015.00042 25767772 PMC4341429

[26] Lowes LE Allan AL. Recent advances in the molecular characterization of circulating tumor cells. Cancers (Basel) (2014) 6:595–624. 10.3390/cancers6010595 24633084 PMC3980613

[27] Baccelli I Schneeweiss A Riethdorf S Stenzinger A Schillert A Vogel V Identification of a population of blood circulating tumor cells from breast cancer patients that initiates metastasis in a xenograft assay. Nat Biotechnol (2013) 31:539–44. 10.1038/nbt.2576 23609047

[28] Chiang AC Massagué J Molecular basis of metastasis. N Engl J Med (2008) 359:2814–23. 10.1056/NEJMra0805239 19109576 PMC4189180

[29] Hanahan D Weinberg RA Hallmarks of cancer: the next generation. Cell (2011) 144:646–74. 10.1016/j.cell.2011.02.013 21376230

[30] Joosse SA Gorges TM Pantel K Biology, detection, and clinical implications of circulating tumor cells. EMBO Mol Med (2015) 7:1–11. 10.15252/emmm.201303698 25398926 PMC4309663

[31] Punnoose EA Atwal SK Spoerke JM Savage H Pandita A Yeh R-F Molecular biomarker analyses using circulating tumor cells. PLoS One (2010) 5:e12517. 10.1371/journal.pone.0012517 20838621 PMC2935889

[32] Yu M Bardia A Wittner BS Stott SL Smas ME Ting DT Circulating breast tumor cells exhibit dynamic changes in epithelial and mesenchymal composition. Science (2013) 339:580–4. 10.1126/science.1228522 23372014 PMC3760262

[33] Krebs MG Metcalf RL Carter L Brady G Blackhall FH Dive C. Molecular analysis of circulating tumour cells-biology and biomarkers. Nat Rev Clin Oncol (2014) 11:129–44. 10.1038/nrclinonc.2013.253 24445517

[34] Kitamura T Qian B-Z Pollard JW Immune cell promotion of metastasis. Nat Rev Immunol (2015) 15:73–86. 10.1038/nri3789 25614318 PMC4470277

[35] Duda DG Duyverman AM Kohno M Snuderl M Steller EJ Fukumura D Malignant cells facilitate lung metastasis by bringing their own soil. Proc Natl Acad Sci U S A (2010) 107:21677–82. 10.1073/pnas.1016234107 21098274 PMC3003109

[36] Uppal A Wightman SC Ganai S Weichselbaum RR An G. Investigation of the essential role of platelet-tumor cell interactions in metastasis progression using an agent-based model. Theor Biol Med Model (2014) 11:17. 10.1186/1742-4682-11-17 24725600 PMC4022382

[37] Wicha MS Hayes DF Circulating tumor cells: not all detected cells are bad and not all bad cells are detected. J Clin Oncol (2011) 29:1508–11. 10.1200/JCO.2010.34.0026 21422428

[38] Grover PK Cummins AG Price TJ Roberts-Thomson IC Hardingham JE. Circulating tumour cells: the evolving concept and the inadequacy of their enrichment by EpCAM-based methodology for basic and clinical cancer research. Ann Oncol (2014) 25:1506–16. 10.1093/annonc/mdu018 24651410

[39] Talasaz AH Powell AA Huber DE Berbee JG Roh K-H Yu W Isolating highly enriched populations of circulating epithelial cells and other rare cells from blood using a magnetic sweeper device. Proc Natl Acad Sci U S A (2009) 106:3970–5. 10.1073/pnas.0813188106 19234122 PMC2645911

[40] Saucedo-Zeni N Mewes S Niestroj R Gasiorowski L Murawa D Nowaczyk P A novel method for the in vivo isolation of circulating tumor cells from peripheral blood of cancer patients using a functionalized and structured medical wire. Int J Oncol (2012) 41:1241–50. 10.3892/ijo.2012.1557 22825490 PMC3583719

[41] Stott SL Hsu C-H Tsukrov DI Yu M Miyamoto DT Waltman BA Isolation of circulating tumor cells using a microvortex-generating herringbone-chip. Proc Natl Acad Sci U S A (2010) 107:18392–7. 10.1073/pnas.1012539107 20930119 PMC2972993

[42] Ozkumur E Shah AM Ciciliano JC Emmink BL Miyamoto DT Brachtel E Inertial focusing for tumor antigen-dependent and -independent sorting of rare circulating tumor cells. Sci Transl Med (2013) 5:179ra47. 10.1126/scitranslmed.3005616 23552373 PMC3760275

[43] Karabacak NM Spuhler PS Fachin F Lim EJ Pai V Ozkumur E Microfluidic, marker-free isolation of circulating tumor cells from blood samples. Nat Protoc (2014) 9:694–710. 10.1038/nprot.2014.044 24577360 PMC4179254

[44] Zieglschmid V Hollmann C Gutierrez B Albert W Strothoff D Gross E Combination of immunomagnetic enrichment with multiplex RT-PCR analysis for the detection of disseminated tumor cells. Anticancer Res (2005) 25:1803–10. 16033103

[45] Harb W Fan A Tran T Danila DC Keys D Schwartz M Mutational analysis of circulating tumor cells using a novel microfluidic collection device and qPCR assay. Transl Oncol (2013) 6:528–38. 10.1593/tlo.13367 24151533 PMC3799195

[46] Alix-Panabieres C Pantel K. Challenges in circulating tumour cell research. Nat Rev Cancer (2014) 14:623–31. 10.1038/nrc3820 25154812

[47] Polzer B Medoro G Pasch S Fontana F Zorzino L Pestka A Molecular profiling of single circulating tumor cells with diagnostic intention. EMBO Mol Med (2014) 6:1371–86. 10.15252/emmm.201404033 25358515 PMC4237466

[48] Barradas AM Terstappen LW. Towards the biological understanding of CTC: capture technologies, definitions and potential to create metastasis. Cancers (Basel) (2013) 5:1619–42. 10.3390/cancers5041619 24305653 PMC3875957

[49] Coumans FA Ligthart ST Uhr JW Terstappen LW. Challenges in the enumeration and phenotyping of CTC. Clin Cancer Res (2012) 18:5711–8. 10.1158/1078-0432.CCR-12-1585 23014524

[50] Lustberg MB Balasubramanian P Miller B Garcia-Villa A Deighan C Wu Y Heterogeneous atypical cell populations are present in blood of metastatic breast cancer patients. Breast Cancer Res (2014) 16:R23. 10.1186/bcr3622 24602188 PMC4053256

[51] Fischer JC Niederacher D Topp SA Honisch E Schumacher S Schmitz N Diagnostic leukapheresis enables reliable detection of circulating tumor cells of nonmetastatic cancer patients. Proc Natl Acad Sci U S A (2013) 110:16580–5. 10.1073/pnas.1313594110 24065821 PMC3799344

[52] Gascoyne PR Noshari J Anderson TJ Becker FF. Isolation of rare cells from cell mixtures by dielectrophoresis. Electrophoresis (2009) 30:1388–98. 10.1002/elps.200800373 19306266 PMC3754902

[53] Vona G Estepa L Beroud C Damotte D Capron F Nalpas B Impact of cytomorphological detection of circulating tumor cells in patients with liver cancer. Hepatology (2004) 39:792–7. 10.1002/hep.20091 14999698

[54] Hou HW Warkiani ME Khoo BL Li ZR Soo RA Tan DS-W Isolation and retrieval of circulating tumor cells using centrifugal forces. Sci Rep (2013) 3:1259. 10.1038/srep01259 23405273 PMC3569917

[55] Allard WJ Matera J Miller MC Repollet M Connelly MC Rao C Tumor cells circulate in the peripheral blood of all major carcinomas but not in healthy subjects or patients with nonmalignant diseases. Clin Cancer Res (2004) 10:6897–904. 10.1158/1078-0432.CCR-04-0378 15501967

[56] Lianidou ES Molecular characterization of circulating tumor cells: Holy Grail for personalized cancer treatment? Clin Chem (2014) 60:1249–51. 10.1373/clinchem.2014.230144 25142245

[57] Neves RP Raba K Schmidt O Honisch E Meier-Stiegen F Behrens B Genomic high-resolution profiling of single CKpos/CD45neg flow-sorting purified circulating tumor cells from patients with metastatic breast cancer. Clin Chem (2014) 60:1290–7. 10.1373/clinchem.2014.222331 25267515

[58] Saliba A-E Saias L Psychari E Minc N Simon D Bidard F-C Microfluidic sorting and multimodal typing of cancer cells in self-assembled magnetic arrays. Proc Natl Acad Sci U S A (2010) 107:14524–9. 10.1073/pnas.1001515107 20679245 PMC2930475

[59] Gallant J-N Matthew EM Cheng H Harouaka R Lamparella NE Kunkel M Predicting therapy response in live tumor cells isolated with the flexible micro spring array device. Cell Cycle (2013) 12:2132–43. 10.4161/cc.25165 23759587 PMC3737315

[60] Lin HK Zheng S Williams AJ Balic M Groshen S Scher HI Portable filter-based microdevice for detection and characterization of circulating tumor cells. Clin Cancer Res (2010) 16:5011–8. 10.1158/1078-0432.CCR-10-1105 20876796 PMC2955786

[61] Issadore D Chung J Shao H Liong M Ghazani AA Castro CM Ultrasensitive clinical enumeration of rare cells ex vivo using a micro-hall detector. Sci Transl Med (2012) 4:141ra92. 10.1126/scitranslmed.3003747 22764208 PMC3603277

[62] Halo TL McMahon KM Angeloni NL Xu Y Wang W Chinen AB NanoFlares for the detection, isolation, and culture of live tumor cells from human blood. Proc Natl Acad Sci U S A (2014) 111:17104–9. 10.1073/pnas.1418637111 25404304 PMC4260589

[63] Dawson S-J Tsui DW Murtaza M Biggs H Rueda OM Chin S-F Analysis of circulating tumor DNA to monitor metastatic breast cancer. N Engl J Med (2013) 368:1199–209. 10.1056/NEJMoa1213261 23484797

[64] Komine K Inoue M Otsuka K Fukuda K Nanjo H Shibata H. Utility of measuring circulating tumor cell counts to assess the efficacy of treatment for carcinomas of unknown primary origin. Anticancer Res (2014) 34:3165–8. 24922689

[65] Harris L Fritsche H Mennel R Norton L Ravdin P Taube S American Society of Clinical Oncology 2007 update of recommendations for the use of tumor markers in breast cancer. J Clin Oncol (2007) 25:5287–312. 10.1200/JCO.2007.14.2364 17954709

[66] Bidard F-C Peeters DJ Fehm T Nolé F Gisbert-Criado R Mavroudis D Clinical validity of circulating tumour cells in patients with metastatic breast cancer: a pooled analysis of individual patient data. Lancet Oncol (2014) 15:406–14. 10.1016/S1470-2045(14)70069-5 24636208

[67] Zhang L Riethdorf S Wu G Wang T Yang K Peng G Meta-analysis of the prognostic value of circulating tumor cells in breast cancer. Clin Cancer Res (2012) 18:5701–10. 10.1158/1078-0432.CCR-12-1587 22908097

[68] Krishnamurthy S Cristofanilli M Singh B Reuben J Gao H Cohen EN Detection of minimal residual disease in blood and bone marrow in early stage breast cancer. Cancer (2010) 116:3330–7. 10.1002/cncr.25145 20564098

[69] Lucci A Hall CS Lodhi AK Bhattacharyya A Anderson AE Xiao L Circulating tumour cells in non-metastatic breast cancer: a prospective study. Lancet Oncol (2012) 13:688–95. 10.1016/S1470-2045(12)70209-7 22677156

[70] Pierga J-Y Bidard F-C Mathiot C Brain E Delaloge S Giachetti S Circulating tumor cell detection predicts early metastatic relapse after neoadjuvant chemotherapy in large operable and locally advanced breast cancer in a phase II randomized trial. Clin Cancer Res (2008) 14:7004–10. 10.1158/1078-0432.CCR-08-0030 18980996

[71] Pierga J-Y Bidard F-C Cropet C Tresca P Dalenc F Romieu G Circulating tumor cells and brain metastasis outcome in patients with HER2-positive breast cancer: the LANDSCAPE trial. Ann Oncol (2013) 24:2999–3004. 10.1093/annonc/mdt348 24013510

[72] Paoletti C Li Y Muniz MC Kidwell KM Aung K Thomas DG Significance of circulating tumor cells in metastatic triple negative breast cancer patients within a randomized, phase II trial: TBCRC 019. Clin Cancer Res (2015) 21(12):2771–9. 10.1158/1078-0432.CCR-14-2781 25779948 PMC5521206

[73] Comen E Norton L Massagué J. Clinical implications of cancer self-seeding. Nat Rev Clin Oncol (2011) 8:369–77. 10.1038/nrclinonc.2011.64 21522121

[74] Zhang L Ridgway LD Wetzel MD Ngo J Yin W Kumar D The identification and characterization of breast cancer CTCs competent for brain metastasis. Sci Transl Med (2013) 5:180ra48. 10.1126/scitranslmed.3005109 23576814 PMC3863909

[75] Charafe-Jauffret E Ginestier C Iovino F Tarpin C Diebel M Esterni B Aldehyde dehydrogenase 1-positive cancer stem cells mediate metastasis and poor clinical outcome in inflammatory breast cancer. Clin Cancer Res (2010) 16:45–55. 10.1158/1078-0432.CCR-09-1630 20028757 PMC2874875

[76] Amato RJ Melnikova V Zhang Y Liu W Saxena S Shah PK Epithelial cell adhesion molecule-positive circulating tumor cells as predictive biomarker in patients with prostate cancer. Urology (2013) 81:1303–7. 10.1016/j.urology.2012.10.041 23622774

[77] Scher HI Jia X de Bono JS Fleisher M Pienta KJ Raghavan D Circulating tumour cells as prognostic markers in progressive, castration-resistant prostate cancer: a reanalysis of IMMC38 trial data. Lancet Oncol (2009) 10:233–9. 10.1016/S1470-2045(08)70340-1 19213602 PMC2774131

[78] Miyamoto DT Sequist LV Lee RJ. Circulating tumour cells-monitoring treatment response in prostate cancer. Nat Rev Clin Oncol (2014) 11:401–12. 10.1038/nrclinonc.2014.82 24821215

[79] Denève E Riethdorf S Ramos J Nocca D Coffy A Daurès J-P Capture of viable circulating tumor cells in the liver of colorectal cancer patients. Clin Chem (2013) 59:1384–92. 10.1373/clinchem.2013.202846 23695297

[80] Cohen SJ Punt CJ Iannotti N Saidman BH Sabbath KD Gabrail NY Prognostic significance of circulating tumor cells in patients with metastatic colorectal cancer. Ann Oncol (2009) 20:1223–9. 10.1093/annonc/mdn786 19282466

[81] Aggarwal C Meropol NJ Punt CJ Iannotti N Saidman BH Sabbath KD Relationship among circulating tumor cells, CEA and overall survival in patients with metastatic colorectal cancer. Ann Oncol (2013) 24:420–8. 10.1093/annonc/mds336 23028040

[82] Lalmahomed ZS Mostert B Onstenk W Kraan J Ayez N Gratama JW Prognostic value of circulating tumour cells for early recurrence after resection of colorectal liver metastases. Br J Cancer (2015) 112:556–61. 10.1038/bjc.2014.651 25562435 PMC4453661

[83] Mostert B Jiang Y Sieuwerts AM Wang H Bolt-de Vries J Biermann K KRAS and BRAF mutation status in circulating colorectal tumor cells and their correlation with primary and metastatic tumor tissue. Int J Cancer (2013) 133:130–41. 10.1002/ijc.27987 23233388

[84] Hou J-M Krebs MG Lancashire L Sloane R Backen A Swain RK Clinical significance and molecular characteristics of circulating tumor cells and circulating tumor microemboli in patients with small-cell lung cancer. J Clin Oncol (2012) 30:525–32. 10.1200/JCO.2010.33.3716 22253462

[85] Maheswaran S Sequist LV Nagrath S Ulkus L Brannigan B Collura CV Detection of mutations in EGFR in circulating lung-cancer cells. N Engl J Med (2008) 359:366–77. 10.1056/NEJMoa0800668 18596266 PMC3551471

[86] Mourad PD Farrell L Stamps LD Chicoine MR Silbergeld DL. Why are systemic glioblastoma metastases rare? Systemic and cerebral growth of mouse glioblastoma. Surg Neurol (2005) 63:511–9. 10.1016/j.surneu.2004.08.062 15936366

[87] Sullivan JP Nahed BV Madden MW Oliveira SM Springer S Bhere D Brain tumor cells in circulation are enriched for mesenchymal gene expression. Cancer Discov (2014) 4:1299–309. 10.1158/2159-8290.CD-14-0471 25139148 PMC4221467

[88] Macarthur KM Kao GD Chandrasekaran S Alonso-Basanta M Chapman C Lustig RA Detection of brain tumor cells in the peripheral blood by a telomerase promoter-based assay. Cancer Res (2014) 74:2152–9. 10.1158/0008-5472.CAN-13-0813 24525740 PMC4144786

[89] Böhm C Wassmann H Paulus W. No evidence of tumour cells in blood of patients with glioma. Mol Pathol (2003) 56:187–9. 10.1136/mp.56.3.187 12782768 PMC1187317

[90] Martens T Matschke J Müller C Riethdorf S Balabanov S Westphal M Skeletal spread of an anaplastic astrocytoma (WHO grade III) and preservation of histopathological properties within metastases. Clin Neurol Neurosurg (2013) 115:323–8. 10.1016/j.clineuro.2012.05.025 22704562

[91] Schuhmann MU Zucht HD Nassimi R Heine G Schneekloth CG Stuerenburg HJ Peptide screening of cerebrospinal fluid in patients with glioblastoma multiforme. Eur J Surg Oncol (2010) 36:201–7. 10.1016/j.ejso.2009.07.010 19674866

[92] Khwaja FW Reed MS Olson JJ Schmotzer BJ Gillespie GY Guha A Proteomic identification of biomarkers in the cerebrospinal fluid (CSF) of astrocytoma patients. J Proteome Res (2007) 6:559–70. 10.1021/pr060240z 17269713 PMC2566942

